# Effect of Orthodontic Tube Base Area and Enamel Sandblasting on Bonding Strength to Enamel: An In Vitro Study

**DOI:** 10.3390/jcm15020579

**Published:** 2026-01-11

**Authors:** Kotryna Osipovė, Livija Maldonytė, Donatas Lukšys, Julius Griškevičius, Rimantas Stonkus, Arūnas Vasiliauskas

**Affiliations:** 1 Department of Orthodontics, Faculty of Odontology, Medical Academy, Lithuanian University of Health Sciences, 50166 Kaunas, Lithuania; livija0217@gmail.com (L.M.); arunas.vasiliauskas@lsmu.lt (A.V.); 2 Department of Biomechanical Engineering, Faculty of Mechanics, Vilnius Gediminas Technical University, 10105 Vilnius, Lithuania; donatas.luksys@vilniustech.lt (D.L.); julius.griskevicius@vilniustech.lt (J.G.); 3 Department of Mechatronics, Robotics, and Digital Manufacturing, Vilnius Gediminas Technical University, Plytines g. 25, 10105 Vilnius, Lithuania; rimantas.stonkus@vilniustech.lt

**Keywords:** base area, enamel preparation, orthodontic tubes, sandblasting, shear bond strength

## Abstract

**Background**: The bond strength of orthodontic tubes to the enamel surface is essential for maintaining appliance stability, especially due to high masticatory forces on molars. Strong adhesion reduces the need for rebonding, shortening treatment time. This study aimed to evaluate the impact of tube base size and enamel sandblasting on bond strength in vitro. **Methods**: Eighty extracted permanent molar teeth were used for this study, divided into four groups of 20 samples each: M—small base tubes (non-sandblasted enamel), SM—small base tubes (sandblasted enamel), T—large base tubes (non-sandblasted enamel), and ST—large base tubes (sandblasted enamel). Shear bond strength was measured using the Mecmesim Multitesters 2.5-I device. Statistical analysis was conducted using IBM SPSS 27.0 software, applying ANOVA and Tukey’s post hoc test. **Results**: The highest bond strength (N) was recorded in the ST group, 85.51 ± 25.04 N, and the lowest in the M group, 50.23 ± 19.76 N. In terms of MPa, the SM group had the highest average value, 11.31 ± 3.57 MPa, while the T group had the lowest, 4.89 ± 1.33 MPa. ANOVA showed a statistically significant effect of tube base size on bond strength (*p* < 0.001), while sandblasting had no significant effect (*p* > 0.05). **Conclusions**: Larger base orthodontic tubes demonstrate stronger adhesion to enamel and are recommended for molars. Sandblasting the enamel does not significantly impact bond strength.

## 1. Introduction

Bond strength between orthodontic attachments and enamel is essential for appliance stability and effective force transmission during treatment. Bond failure increases chair time and treatment duration and may cause patient discomfort and soft-tissue irritation [1,2,3].

This issue is particularly relevant for molar tubes, which are exposed to higher masticatory loads than anterior attachments because archwires are directly engaged and forces are transferred to posterior teeth during function [4,5]. Therefore, sufficient bond strength of molar tubes is critical to minimize detachment.

At the same time, the demand for less visible and more comfortable appliances has promoted smaller attachment designs. However, reducing the base area may compromise bonding performance by limiting the available bonding surface [6,7].

Bonding strength of braces depends on variety of factors, including type of adhesive or utilized bonding system, preparation of tooth surface, design of bracket base, defects in the enamel or dentin, patient’s age, oral hygiene, bite characteristics, and even whitening procedures performed prior to orthodontic treatment [1].

To improve bond strength of orthodontic appliances, various surface preparation methods are used. Sandblasting is most frequently used. Sandblasting is an air abrasion procedure that uses fine aluminum oxide particles. This technique can be applied to both—the base of the bracket or tube itself to increase its roughness, and to the surface of the tooth enamel [8,9]. By creating an irregular and textured surface, the mentioned technique allows orthodontic adhesives to penetrate the enamel better and form a stronger bond. Surface modification increases contact area of the adhesive and its mechanical adhesion to microstructures, which has a positive effect on the overall bond strength. Studies show that sandblasting can significantly increase bond strength compared to untreated enamel or bracket surfaces [8].

However, evidence regarding the effectiveness of enamel sandblasting remains contradictory. Canay et al. reported improved retention after enamel air abrasion, whereas Daratsianos et al. and Robles-Ruíz et al. reported no significant improvement in shear bond strength when enamel sandblasting was performed in addition to phosphoric-acid etching [10,11,12]. Although smaller orthodontic attachments are increasingly preferred for aesthetic and comfort reasons, evidence on how reduced tube base area affects bond strength—particularly for molar tubes—remains limited. Therefore, this in vitro study aims to comprehensively evaluate the influence of both reduced wire base size and enamel sandblasting on the final bond strength, with the aim of determining whether sandblasting can compensate for the reduced bonding area and maintain a clinically acceptable level of bond strength. The null hypotheses were: (H0a) orthodontic tube base area has no significant effect on bonding strength to enamel, and (H0b) additional enamel sandblasting prior to phosphoric-acid etching has no significant effect on bonding strength between orthodontic tubes and enamel.

## 2. Materials and Methods

### 2.1. Sample Size

An a priori sample size calculation was performed using the G*Power (GIGA) calculator, version 3.1.9.7 (Heinrich Heine University Düsseldorf, Düsseldorf, Germany), for a one-way ANOVA design (four groups), with shear bond strength as the primary outcome. The calculation assumed 80% power and α = 0.05; a medium-to-large effect size was prespecified (Cohen’s f = 0.40), resulting in a minimum required sample size of *n* = 32 (8 specimens per group). To increase reliability and reduce random variation, we included 80 specimens (20 per group) [13].

### 2.2. Selection of Teeth for the Study

Study protocol was prepared prior to the study. On 26 February 2025, approval was received from the LSMU Bioethics Centre Committee (No. 2025-BEC2-0315). Teeth used in the study were permanent molars removed for surgical, periodontal, or orthodontic reasons at the LSMU Faculty of Medicine’s Clinic of Maxillofacial Surgery. All patients were informed about the study before surgical interventions and were also provided with an information form about the study, and obtained written consent to participate. The study included molars that were not affected by caries or fluorosis, had not been restored, and had no enamel fissures on the buccal surface.

### 2.3. Tooth Preparation and Bonding of Orthodontic Tubes

Eighty extracted permanent molars that met the inclusion criteria were used for the study and randomly divided into four groups.

(1) M—orthodontic tubes with a small base area bonded to teeth with an enamel surface that was not sandblasted.

(2) SM—orthodontic tubes with a small base area bonded to teeth with an enamel surface that was sandblasted.

(3) T—orthodontic tubes with a large base area bonded to teeth with an enamel surface that was not sandblasted.

(4) ST—orthodontic tubes with a large base area bonded to teeth with an enamel surface that was sandblasted.

#### 2.3.1. Tooth Preparation

The extracted teeth were initially kept in Gigasept Instru AF solution (Schülke & Mayr GmbH, Norderstedt, Germany) for 15 min and subsequently rinsed under running water for one minute. Prior to the study, extracted human teeth were stored in 0.9% sodium chloride solution at room temperature for up to two months before the bonding procedures [12].

Before bonding, soft tissue residues as well as hard and soft deposits were removed mechanically. The tooth surfaces were cleaned for 30 s with fluoride-free Cleanic polishing paste (KerrHawe SA, Bioggio, Switzerland) using a polishing brush and a straight tip (5000 rpm). The polishing paste residue was washed off with water for 30 s, and the tooth surfaces were dried with compressed air for 10 s.

#### 2.3.2. Bonding of Orthodontic Tubes

All orthodontic tubes were bonded using GC Ortho Connect (GC Corporation, Tokyo, Japan), a one-step light-curing adhesive that does not require a primer. The sandblasting procedure applied to the SM and ST groups was performed using Groman EtchMaster 50 μm aluminum oxide sand (Fordentist, Kaunas, Lithuania) at a pressure of 80 psi for 5 s, maintaining a distance of 10 mm and an angle of 45°, in accordance with previously published experimental protocols [14,15]. Sandblasting was performed at the Department of Orthodontics of the Lithuanian University of Health Sciences. Orthodontic tubes were bonded according to the following procedure: 

Group M (without enamel sandblasting, small base).

The enamel was etched with 37% orthophosphoric acid gel “i-GEL” (G&H Orthodontics, Franklin, IN, USA) for 40 s, rinsed with water for 30 s, and air-dried until matte. “Mini Buccal Tubes” (Forestadent Bernhard Förster GmbH, Pforzheim, Germany; base area 5.5 mm^2^) were placed, excess adhesive removed, and polymerized with a VALO Ortho Cordless LED lamp (Ultradent Products, South Jordan, UT, USA) for 40 s (10 s per side, 1 mm distance).

SM group (with sandblasting, small base).

Before etching, the tooth enamel was additionally sandblasted with Groman EtchMaster 50 μm aluminum oxide sand at 80 psi for 5 s, 10 mm distance, and a 45° angle, following published protocols for enamel surface preparation prior to bonding [14,15], then rinsed with water (30 s) and dried until the surface became matte. The enamel was then etched with i-GEL 37% orthophosphoric acid gel for 40 s, rinsed again (30 s) and dried. “Mini Buccal Tubes” orthodontic tubes with a base area of 5.5 mm^2^ were used. They were bonded in accordance with the M group methodology.

Group T (without enamel sandblasting, large base).

The bonding procedure is identical to group M, but large-base orthodontic tubes “Tulip Tubes” (Forestadent Bernhard Förster GmbH, Pforzheim, Germany) with a base area of 17 mm^2^ were used.

ST group (with enamel sandblasting, large base).

The bonding procedure is identical to that for the SM group, but large-base orthodontic tubes “Tulip Tubes” with a base area of 17 mm^2^ are used.

The orthodontic tubes were positioned in the center of the buccal surface of the tooth crowns, excess adhesive removed using dental probe.

### 2.4. Fixing of Specimens and Measurement of Bond Strength

**Figure 1:**
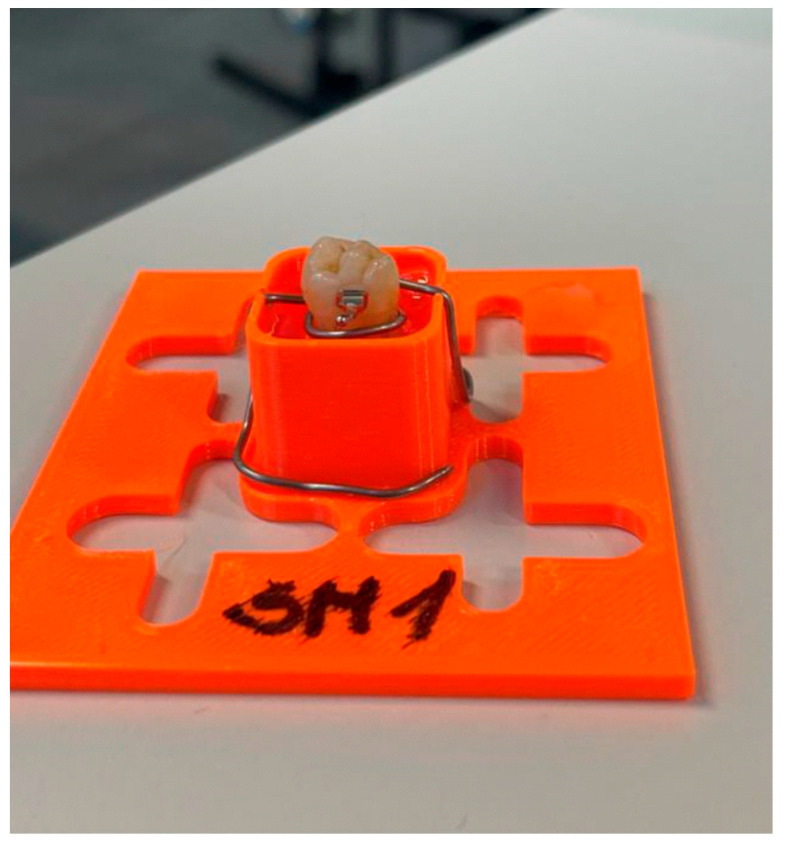
Prepared tooth specimen.

Each tooth was fixed up to the neck in identical plastic holders, which were designed specifically for the study and manufactured using a Zortrax M300 Plus 3D printer (Zortrax S.A., Olsztyn, Poland) from Devil Design PLA plastic (Devil Design, Żory, Poland, 1.75 mm). Transparent epoxy resin Epoxidharz L with hardener Härter L (R&G Faserverbundwerkstoffe GmbH, Waldenbuch, Germany) was used to fix the teeth in the mold. The epoxy resin was prepared in mixing ratio of 100:30 (mixing time-3 min) and poured into the mold for fixing the specimens. The teeth were left at room temperature (22 °C) for 24 h to ensure the stability of the specimens throughout the study. A prepared tooth specimen was considered suitable if it met the following criteria: stable tooth position, no tilting of the tooth axis, completely hardened epoxy resin, exposed tooth crown. Prepared tooth specimen (Figure 1). To minimize specimen misalignment, each tooth was aligned visually in the holder using the flat reference surfaces of the mold before epoxy curing. After curing, specimens were re-inspected; visibly tilted samples were excluded and remounted. Tilt angle was not measured quantitatively.

The samples were fixed in a machine. A Mecmesim Multitesters 2.5-I device (Mecmesim Limited, Horsham, UK) was used to evaluate the force required to remove the orthodontic tubes. The test was performed in the laboratory of the Department of Biomechanical Engineering at Vilnius University of Technology.

The device was connected to a computer, which recorded the force required to remove the orthodontic tubes until they were completely detached with a constant speed of 0.1 mm/s. The adhesion of the orthodontic tubes was assessed based on the removal force (N).

### 2.5. Statistical Data Analysis

The collected data was recorded in real time using EMPEROR software, version 1.18 (Mecmesim Limited, UK), which recorded changes in the force required to remove the orthodontic tubes. The test data was exported to the Microsoft Excel 2021 computer program. Statistical data analysis was performed using SPSS IBM 27.0 (Statistical Package for Social Sciences) software. Quantitative data were analyzed according to the following descriptive statistical criteria: mean, standard deviation, first (Q1) and third (Q3) quartiles, minimum and maximum values.

Four independent groups (M, T, SM, ST) were compared in the study, and the Shapiro–Wilk test was used to test the assumption of normality. Homogeneity of variance was assessed using the Levene test. Both assumptions were confirmed (*p* > 0.05). A one-way ANOVA was performed to assess the differences. The ANOVA results showed a statistically significant difference between at least one of the groups studied (*p* < 0.05), so a post hoc Tukey test was performed to determine which specific groups differed from each other.

Statistical significance was assessed using a significance level of α = 0.05. Differences between the study groups were considered statistically significant when *p* < 0.05.

## 3. Results

### 3.1. Bond Strength (N)

The aim of this in vitro study was to evaluate the bond strength of different orthodontic tubes to tooth enamel using various surface preparation techniques. Samples in which the tubes detached at a force lower than 10 N (N < 10) were excluded from the study, as this was considered to be complete detachment of the tube without actual adhesion—these results did not reflect the actual strength of the bond [6,16]. These specimens were replaced to maintain *n* = 20 per group. Four groups were analyzed in the study: M, T, SM, and ST. Each group consisted of 20 samples, whose bond strength was measured in newtons (N) (Table 1).

Regardless the preparation method of enamel and tube area, total average bond strength in newtons (N) in all studied cases was 70.77 N. Statistical results show that the highest average bond strength was demonstrated by the ST group 85.51 ± 25.04 N, followed by group T with 83.14 ± 22.62 N, group SM with 62.21 ± 19.64 N, and the lowest was demonstrated by group M with 50.23 ± 19.76 N. The minimum debonding force was recorded in group M at 21.4 N, and the maximum in group ST at 130.7 N.

ANOVA analysis revealed a statistically significant effect of groups on bond strength (*p* < 0.001), indicating that the area of the orthodontic tube or the method of enamel preparation has a significant effect on bond strength. In order to determine which specific groups show statistically significant differences, the following was calculated.

(1) T and M: Group T showed significantly higher debonding force (*p* < 0.001).
(2) Groups T and SM: Group T had significantly higher debonding force (*p* < 0.05).
(3) ST and M groups: The ST group showed significantly greater debonding strength (*p* < 0.001).
(4) ST and SM groups: The ST group had significantly greater debonding strength (*p* < 0.01).

**Figure 2:**
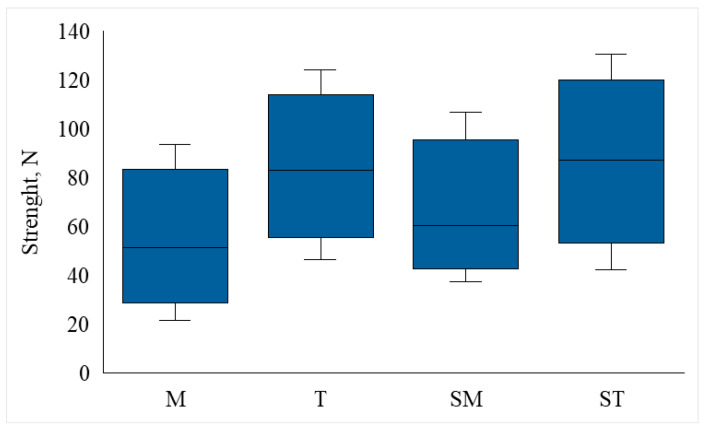
Comparison of removal forces (N) between different groups. M, small orthodontic tubes; T, large orthodontic tubes; SM, small orthodontic tubes bonded to sandblasted enamel; ST—large orthodontic tubes bonded to sandblasted enamel.

The comparison of removal forces (N) between different groups is depicted in (Figure 2). The mean required removal force (N) in group M 50.23 ± 19.76 N was statistically significantly lower than in group T 83.14 ± 22.62 N (*p* < 0.001). This tendency was also observed when comparing large (ST) and small (SM) tubes bonded to sandblasted enamel—the larger orthodontic tube base resulted in a stronger debonding force (N) to enamel: 85.51 ± 25.04 N and 62.21 ± 19.64 N, respectively.

Large-base orthodontic tubes bonded to sandblasted enamel (ST) had a higher average bond strength of 85.51 ± 25.04 N compared to large tubes bonded to unsandblasted enamel (group T) of 83.14 N, yet Tukey’s test showed that this difference was not statistically significant (*p* > 0.05), therefore enamel sandblasting does not significantly increase bond strength (N).

The SM group (small area orthodontic tubes with sandblasted enamel) has a slightly higher mean debonding force of 62.21 ± 19.64 N than the M group 50.23 ± 19.76 N. However, Tukey’s test showed that the difference between the M and SM groups is not statistically significant (*p* > 0.05). The following statistical values are presented in the graphs: mean, first quartile (Q1), third quartile (Q3), median, minimum value, maximum value, value less than ½ interquartile difference from the third quartile.

### 3.2. Shear Bond Strength (MPa)

Following further data analysis, shear bond strength values were expressed in megapascals (MPa) to more accurately represent the force distribution over the contact area. Shear bond strength (MPa) was calculated using the relation 1 MPa = 1 N/mm^2^, where mm^2^ corresponds to the tube base surface area. The base areas were 5.5 mm^2^ for small tubes (M, SM) and 17 mm^2^ for large tubes (T, ST). Shear bond strength results for each group are summarized in (Table 2).

**Figure 3:**
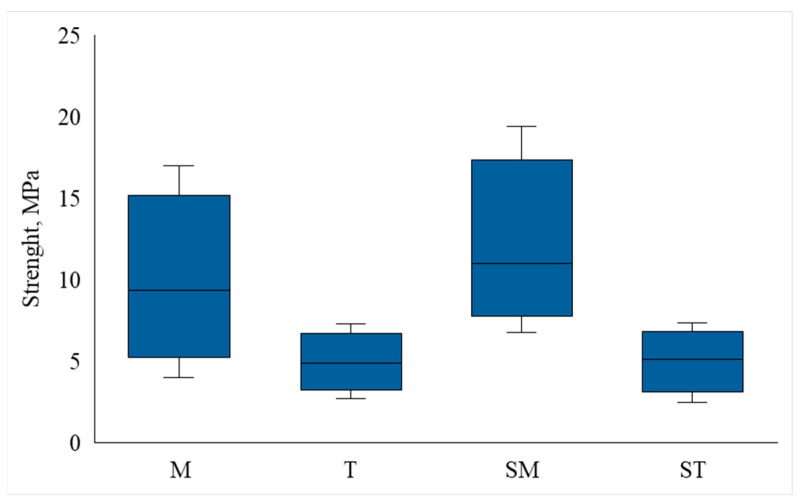
Distribution of shear bond strength (MPa) in different test groups. M, small orthodontic tubes; T, large orthodontic tubes; SM, small orthodontic tubes bonded to sandblasted enamel; ST—large orthodontic tubes bonded to sandblasted enamel.

Observed shear bond strength rates (MPa) between different groups indicated that overall average shear bond strength was 7.09 MPa. These strength rates significantly vary (SD 3.03) depending on tube size and preparation type of enamel (Figure 3)

Statistical results reveal that the highest average shear bond strength per unit area was demonstrated by group SM at 11.31 ± 3.57 MPa, followed by group M at 9.13 ± 3.59 MPa, group ST at 5.03 ± 1.47 MPa, and the lowest value was recorded in group T (4.89 ± 1.33 Mpa). The minimum shear bond strength was recorded in group T (2.72 MPa), while the maximum was indicated in group SM (19.40 MPa) (Table 2).

The average shear bond strength per unit area was demonstrated by group T 4.89 ± 1.33 MPa. The minimum shear bond strength was registered in group T (2.72 MPa), and the maximum in group SM (19.40 MPa) (Table 2).

ANOVA analysis revealed a statistically significant effect of groups on the shear bond strength (*p* < 0.001). To determine between which specific groups there were statistically significant differences, an additional pairwise analysis was performed using the Tukey test:

(1) Groups M and T: Group M showed significantly higher shear bond strength. (*p* < 0.001).
(2) Groups M and ST: Group M had significantly higher shear bond strength. (*p* < 0.001).
(3) SM and T groups: The SM group showed significantly greater shear bond strength. (*p* < 0.001).
(4) SM and ST groups: The SM group had significantly greater shear bond strength (*p* < 0.001).

## 4. Discussion

Bond strength of orthodontic brackets and tubes must ensure sufficient adhesion throughout treatment and safe, easy removal of the brackets or tubes at the end of treatment. Excessive adhesion can cause microcracks in the enamel, while insufficient adhesion may lead to bracket or tube debonding. Thus, optimal balance between adhesion strength and easy removal is essential [17,18,19,20].

Size of tube base area can affect bond strength between orthodontic tubes and tooth enamel the most. Tubes with a large base area exhibit robust adhesion to enamel, since larger contact area ensures great mechanical stability. It is noteworthy that most prior studies in the literature are conducted with hooks and brackets [2,6,18], whereas our study examined molars and tubes, which experience the greatest functional load during chewing [5,21,22]. This confirms once again that sufficient bond strength is particularly important for tubes that are bonded to molars. The standard deviation (SD) differed between groups: the highest SD was in the ST group (25.04), while in the SM (19.64) and M (19.76) groups it was significantly lower, indicating less variation in results and more stable adhesion of the tube to the tooth surface, regardless of individual tooth characteristics. These results are consistent with previous studies confirming that large base brackets have stronger adhesion (N) to tooth enamel. Pham D. describes that the highest bond strength N was recorded for soccer ball and flower-shaped brackets with a base area of 32.26–40.58 mm^2^, compared to a rectangular bracket with a smaller base width of 17.63 mm^2^. Meanwhile, the analysis of the shear bond strength (MPa) showed the opposite trend—the highest MPa value was recorded for rectangular brackets, and the lowest for football and flower-shaped brackets (*p* < 0.01) [6]. The difference arises because the MPa value represents the force per unit area, hence, brackets with larger bases, despite their higher adhesion force (N), display lower MPa values as the applied force is spread over a greater surface area. From a clinical perspective, shear bond strength (MPa) is particularly informative because it expresses the debonding force per unit bonding area. Therefore, MPa may better reflect clinical performance when comparing attachments with different base sizes, while debonding force (N) mainly reflects the overall removal force.

The results of our study verify this pattern: the SM 11.31 ± 3.57 MPa and M 9.13 ± 3.59 MPa groups demonstrated significantly higher shear bond strength compared to the ST 5.03 ± 1.47 MPa and T 4.89 ± 1.33 MPa groups. This confirms that small base tubes have greater adhesion strength per unit area compared to large base tubes. This difference can be explained by the fact that a smaller base area creates a greater concentration of force at a single point, so the force acting on the areas is more concentrated [6,18,20].

Our results showed that additional enamel sandblasting before conventional phosphoric-acid etching had no statistically significant effect on debonding force (N) or shear bond strength (MPa) (*p* > 0.05); therefore, the null hypothesis (H0b) was accepted. These findings are consistent with reports indicating that enamel sandblasting performed prior to acid etching does not necessarily improve bond strength [20,21].

Importantly, this result does not imply that sandblasting is universally ineffective; rather, its benefit appears to be substrate- and protocol-dependent. Sandblasting has also been shown to be beneficial for bonding to certain restorative materials, such as zirconia crowns or composite restorations [18,23,24,25]. Therefore, under the conditions of the present study, additional enamel sandblasting before phosphoric-acid etching is not recommended solely to increase bond strength on intact enamel. In contrast, when bonding to restorative substrates sandblasting may be considered as part of the surface-conditioning protocol according to the substrate-specific evidence and the manufacturer’s recommendations.

In summary, the results of our study have shown that the base area of orthodontic tubes has a significant influence on bond strength, which is essential for ensuring the long-term stability and functionality of orthodontic appliances. Large tubes, such as Tulip Tubes, have good bond strength to withstand chewing forces (60–80 N), but the shear bond strength (MPa) does not exceed the recommended limit of 8 MPa, thus reducing the risk of enamel damage [20,21]. This balance is essential to ensure not only the stability of orthodontic appliances during treatment, but also their safe removal at the end of treatment. Although in our study, sandblasting natural enamel before acid etching had no significant effect, literature data show that this method can be effective when bonding orthodontic tubes to restored surfaces [23,24,25]. In clinical practice, it is important to assess the individual conditions of the patient and to choose the most appropriate surface preparation method to ensure optimal adhesion, long-term treatment effectiveness, and to avoid complications.

Most teeth used in the study were wisdom molars, whereas in clinical practice, tubes are most often bonded to first or second molars, hence differences in enamel structure and load distribution may have influenced the results. It should also be considered that in vitro studies cannot accurately replicate the conditions in the oral cavity or simulate the effects of long-term mechanical load, temperature fluctuations, saliva, and other biological factors. Morphological and anatomical features of teeth, such as the curvature of the enamel surface or the individual shape of the crown, may affect the position of the tube and the quality of adhesion. The use of third molars may affect the external validity and comparability of the results with clinical molar bonding because third molars differ from first and second molars in several clinically relevant aspects. Enamel structure in third molars can be more variable due to differences in mineralization, eruption status, and developmental conditions, which may influence adhesive penetration and bond strength. Additionally, third molars often present different crown morphology and enamel curvature, potentially affecting tube positioning, stress distribution, and measured bond strength values.

From a clinical perspective, orthodontic tubes are most bonded to first and second molars, which are exposed to higher and more consistent masticatory forces and function under different biomechanical conditions than third molars. Therefore, bond strength values obtained from third molars may not fully replicate the clinical loading patterns, failure modes, or long-term performance observed in routine orthodontic treatment.

A major limitation of this in vitro study is that failure mode (fracture pattern/ARI) was not assessed because post-debonding microscopic evaluation was not performed. Future studies should include standardized failure mode assessment (e.g., ARI scoring under magnification) to better interpret the clinical implications of bond strength findings.

## 5. Conclusions

In conclusion, tube base area significantly influenced bonding outcomes. Large-base tubes required higher debonding (removal) forces (N), whereas small-base tubes demonstrated higher shear bond strength (MPa), indicating greater adhesion strength per unit area. The highest mean shear bond strength was observed in the small-base sandblasted group (SM), and it was significantly higher than both large-base groups (T and ST) (*p* < 0.001). Additional enamel sandblasting before phosphoric-acid etching did not significantly affect bond strength under the conditions tested (*p* > 0.05).

## Figures and Tables

**Table 1:** Debonding force results for different groups (N).

Group	Sample Size (*n*)	Mean (SD) N	Minimum Value N	Maximum Value N	Median N
M	20	50.23 ± 19.76	21.4	93.7	52.55
T	20	83.14 ± 22.62	46.2	124.1	82.85
SM	20	62.21 ± 19.64	37.3	106.7	58.85
ST	20	85.51 ± 25.04	42.2	130.7	88.85

M, small orthodontic tubes; T, large orthodontic tubes; SM, small orthodontic tubes bonded to sandblasted enamel; ST—large orthodontic tubes bonded to sandblasted enamel; *n*, number of samples; SD, standard deviation; N, unit of measurement used to express bond strength between orthodontic tubes and the enamel surface.

**Table 2:** Shear bond strength results (MPa) for different groups.

Group	Sample Size (*n*)	Mean (SD) (MPa)	Minimal Value (MPa)	Maximum Value (MPa)	Median (MPa)
M	20	9.13 (3.59)	3.89	17.04	9.55
T	20	4.89 (1.33)	2.72	7.30	4.87
SM	20	11.31 (3.57)	6.78	19.40	10.70
ST	20	5.03 (1.47)	2.48	7.39	5.23

M, small orthodontic tubes; T, large orthodontic tubes; SM, small orthodontic tubes bonded to sandblasted enamel; ST—large orthodontic tubes bonded to sandblasted enamel; *n*, number of samples; SD, standard deviation; MPa, unit of measurement, which is more accurate to measure adhesion strength than newton (N) and was used to represent the force distribution over the contact area between orthodontic tubes and enamel surface.

## Data Availability

The data presented in this study are available on request from the corresponding author.

## Abbreviations

The following abbreviations are used in the manuscript:

M	small base tubes (non-sandblasted enamel)
SM	small base tubes (sandblasted enamel)
T	large base tubes (non-sandblasted enamel)
ST	large base tubes (sandblasted enamel).
MPa	megapascals
N	newtons
*n*	sample size
p	level of significance
LED	light-emitting diode
psi	pounds per square inch
SD	standard deviation
3D	three-dimensional
LSMU	Lithuanian University of Health Sciences
